# Different Antibody Response against the Coxsackievirus A16 VP1 Capsid Protein: Specific or Non-Specific

**DOI:** 10.1371/journal.pone.0162820

**Published:** 2016-09-13

**Authors:** Yingying Ding, Zhihong Wang, Xi Zhang, Zheng Teng, Caixia Gao, Baohua Qian, Lili Wang, Jiaojiao Feng, Jinhong Wang, Chunyan Zhao, Cunjiu Guo, Wei Pan

**Affiliations:** 1 Department of Medical Microbiology and Parasitology, School of Basic Medicine, Second Military Medical University, Shanghai, China; 2 Department of Clinical Laboratory, the Second Affiliated Hospital of Shanxi Medical College, Taiyuan, China; 3 Department of Infectious Disease Control and Prevention, Shanghai Municipal Center for Disease Control and Prevention, Shanghai, China; 4 Department of Blood Transfusion, Changhai Hospital, Second Military Medical University, Shanghai, China; Universidad Autonoma de Madrid Centro de Biologia Molecular Severo Ochoa, SPAIN

## Abstract

Coxsackievirus A16 (CA16) is one of the major causative agents of hand, foot, and mouth disease worldwide. The non-neutralizing antibody response that targets CA16 VP1 remains poorly elucidated. In the present study, antibody responses against CA16 VP1 in Shanghai blood donors and Shanxi individuals were analyzed by ELISA and inhibitory ELISA using five CA16 VP1 antigens: VP1_1-297_, VP1_41-297_, VP1_1-60_, VP1_45-58_ and VP1_61-297_. The correlation coefficients for most of the reactions against each of the five antigens and the inhibition of the anti-CA16 VP1 antibody response produced by the various antigens were higher in Shanghai blood donors compared to those in Shanxi individuals. VP1_1-297_ and VP1_41-297_ strongly inhibited the anti-CA16 VP1 response in serum samples from both populations, while VP1_45-58_ and VP1_61-297_ intermediately and weakly inhibited the anti-CA16 VP1 response, respectively, in only Shanghai group. A specific type of inhibition (anti-CA16 VP1 was completely inhibited by both VP1_1-60_ and VP1_41-297_) characterized by high neutralizing antibody titers was identified and accounted for 71.4% of the strongly reactive samples from the Shanghai group. These results indicate that the Shanghai blood donors exhibited a consistent and specific antibody response, while the Shanxi individuals showed an inconsistent and non-specific antibody response. These findings may improve the understanding of host humoral immunity against CA16 and help to identify an effective approach for seroepidemiological surveillance and specific diagnosis of CA16 infection based on normal and competitive ELISA.

## Introduction

Hand, foot, and mouth disease (HFMD) is a common infectious illness that usually affects children, particularly those less than 5 years old [1–3]. Since the first case was reported in 1969, HFMD has continued to spread globally and is a continuing threat to public health [4–6]. Several large outbreaks of HFMD were reported in eastern and southeastern Asian countries and regions during the late 20th century [7–10]. Since 2008, a dramatic increase in the prevalence of HFMD has been reported in mainland China [1, 11–13]. Coxsackievirus A16 (CA16) and enterovirus 71 (EV71) are the major etiological agents of HFMD. The isolation of an increasing number of enteroviruses (EV, a genus in the *Picornaviridae* family) has allowed their phylogenic classification into 12 species, namely, enterovirus A, B, C, D, E, F, G, H and J (*EV A–J*) and rhinovirus A, B and C. In addition to CA2-8, A10, A12, A14 and EV71, CA16 has been classified as an *EV A* species based on its genome sequence [14, 15].

The host humoral immune response plays a key role in controlling and the pathophysiology of viral infections. Studies concerning host humoral immune responses against CA16, EV71 and other enteroviruses have been primarily based on assessments of neutralizing antibodies. Approximately half of neonates (50.0–57.6%) obtain protective neutralizing antibodies from their mothers; however, up to 90.0–98.0% of infants lose these neutralizing antibodies within 6–7 months, thereby becoming vulnerable to CA16 and EV71 infections. The seroprevalences of CA16- and EV71-neutralizing antibodies peak (80.0–100.0%) in children from 1 to 6 years of age, indicating that most primary infections are acquired during early childhood. Adults maintain a high seroprevalence of neutralizing antibodies (40.0–85.3%) with a low incidence of HFMD [5, 16–22].

Both members of the enterovirus family, CA16 and EV71 are each composed of 60 copies of four capsid proteins (VP1, VP2, VP3 and VP4) that form a symmetrical icosahedral structure. The viral capsid proteins VP1, VP2 and VP3 all contain beta-sandwich “jelly-roll” folds and are exposed on the virus surface, while the smallest protein (VP4) is arranged inside the icosahedral lattice [23–25]. Of the viral protein, VP1 is the most highly exposed and has been suggested to play an important role in viral pathogenesis and virulence [26–28]. The neutralizing epitopes on the capsids of CA16 and EV71 have been identified [29–34], but these epitopes only cover a small part of the exposed capsid and may contain only a small proportion of targets for host antibodies. Our previous study characterized host antibody responses against the EV71 capsid and consistently found that the responses were predominantly directed against VP1, particularly to epitopes based on the common enterovirus cross-reactive sequence (CECRS) [35]. This type of antibody response (representing the major host antibody response to EV71 infection) is completely different from the neutralizing antibody response and is named the non-neutralizing antibody response. During this response, cross-reactions between VP1 of EV71 and VP1 variants of closely related viruses are likely. Moreover, the serological prevalences of anti-VP1 for the EV-A species CA5, CA6, CA16 and EV71; the EV-B species CB3; and the EV-C species Poliovirus 1 (PV1) in were determined, and all reactions were significantly correlated at different levels, which were approximately proportional to their sequence similarities [36]. Based on these findings, we proposed the hypothesis that the non-neutralizing antibody response that targets CA16 VP1 should involve both the antibody response that is elicited by infection with CA16 (i.e., a specific antibody response) and the cross-reactive antibody response elicited by infection with CA16-related enteroviruses such as EV71, CA6 and CA10 (i.e., a non-specific antibody response). Whether these two types of antibody response can be delineated remains unknown. To address this issue, in the current study, various CA16 VP1 antigens were used to characterize non-neutralizing antibody responses against CA16 in Shanghai blood donors and Shanxi individuals using ELISA and inhibitory ELISA. The results showed that antibody responses against CA16 VP1 clearly differ between these two groups might represent specific and non-specific antibody responses.

## Materials and Methods

### Ethics statement

The study protocol was approved by the Ethics Committee of the Second Affiliated Hospital of Shanxi Medical College, Taiyuan, China. All experiments were performed in accordance with approved guidelines of the Ethics Committee of the Second Affiliated Hospital of Shanxi Medical College and Second Military Medical University. Written informed consent was obtained from the participants in the study.

### Clinical samples

Ninety-five serum specimens from outpatients with chronic diseases without symptoms of fever or herpes were collected from the Second Affiliated Hospital of Shanxi Medical College, forming the Shanxi group. The mean age of the Shanxi group was 61.2 years old. Informed consent was obtained from each of the participants prior to blood collection. Additionally, 142 serum specimens were collected from healthy blood donors at Changhai Hospital in Shanghai, China, forming the Shanghai group. The relevant information for each of the two hundred and thirty-seven samples is provided in S1 Table. All samples were stored at -80°C in 1.5 ml aliquots.

### Cells and virus strains

An RD (rhabdomyosarcoma) cell line was obtained from the American Type Culture Collection (ATCC number CCL-136) and maintained in Dulbecco’s modified Eagle medium (DMEM, HyClone, Logan, UT, USA) supplemented with 2.0% or 10.0% fetal bovine serum (FBS) (Gibco, Grand Island, NY, USA) and 2 mmol/L L-glutamine at 37°C with 5% CO2. The CA16 virus strain which was isolated from a clinical specimen in Fuyang, Anhui, China in 2008 (GenBank: EU812514.1), was kindly provided by the Shanghai Municipal Center for Disease Control and Prevention.

### Vectors, bacterial strains and reagents

The prokaryotic expression plasmids pET-32a and pET21b and two *E*. *coli* host strains (BL21 (DE3) and Top10) were purchased from Novagen (Darmstadt, Germany). HRP-LD5 consists of HRP conjugated to LD5, which is a novel evolved immunoglobulin-binding molecule (NEIBM) with a characteristic structure composed of an alternating B3 domain from Finegoldia magna protein L and a D domain from staphylococcal protein A. This configuration creates synergistic double-binding sites for fragment of antigen binding (Fab) VH3 and Vk regions and IgG Fc [37]. HRP-LD5 shows high binding affinity for IgM, IgG and IgA [38].

### Cloning of CA16 VP1 gene fragments and construction of expression plasmids

The amino acid sequence of CA16 VP1 (human coxsackievirus A16 capsid protein VP1) was obtained from GenBank (GenBank accession number EU812514.1). The DNA sequence encoded by CA16-VP1 was synthesized using sequential OE-PCR [39] and T/A-cloned into a pMD18-T vector (Takara). The recombinant CA16-VP1 plasmid (VP1-pMD18-T) was used as a template to amplify VP1_1-297_ (full-length VP1), VP1_41-297_, VP1_61-297_, VP1_1-60_ and VP1_45-58_ using the primer pairs uVP1/dVP1, uVP1-1/dVP1-1, uVP1-2/dVP1-1, dVP1-3/uVP1-3 and uVP1-4/dVP1-4 (S2 Table), respectively. Additionally, uVP1 contains *Nde* I restriction sites, dVP1 contains *Xho* I restriction sites, uVP1-1 and uVP1-2 contain *Nco* I restriction sites, dVP1-1 contains *Sac* I restriction sites, uVP1-3 and uVP1-4 contain *Bam*H I restriction sites (underlined), and dVP1-1 contains *Hin*d III restriction sites. The PCR products of CA16 VP1_41-297_, VP1_61-297_, VP1_1-60_ and VP1_45-58_ were inserted into the cloning sites of a pET32a vector under the control of the T7 promoter, and the PCR product of CA16 VP1_1-297_ was inserted into the cloning sites of a pET21b vector. A His tag was added at the N-terminus or C-terminus of each target protein to form a fusion protein. These expression plasmids were individually verified by sequencing.

### Expression and purification of recombinant full-length CA16 VP1 and truncated VP1 proteins

*E*. *coli* BL21 (DE3) competent cells transformed with the CA16 capsid protein VP1 and the four truncated CA16 VP1 expression plasmids were cultured in Luria broth (LB) medium supplemented with 100 μg/ml ampicillin (for *E*. *coli* transformed with the pET32a or pET21b vectors) at 37°C in a shaker at 200 rpm. When the OD_600_ of the culture reached 0.6, IPTG was added to a final concentration of 1 mmol/L. After additional incubation for 2–3 h at 37°C, bacterial pellets were harvested by centrifugation at 6000 × g for 20 min and the target proteins were detected using SDS-PAGE. The proteins were purified using Ni-NTA resin (Qiagen, Hilden, Germany). The purified proteins were immediately aliquoted and stored at -80°C prior to analysis.

### Indirect ELISA of antibodies against full-length and truncated CA16 VP1 proteins

Detection of anti-CA16 VP1_1-297_, VP1_41-297_, VP1_61-297_, VP1_1-60_ and VP1_45-58_ antibody responses was performed using ELISA with NEIBM-derived conjugated HRP-LD5 (NEIBM-ELISA) as previously described [35]. Briefly, 96-well ELISA plates (Nunc, Rochester, NY, USA) were coated with 1.0 μg of CA16 VP1_1-297_, VP1_41-297_, VP1_61-297_, VP1_1-60_ or VP1_45-58_ in 0.1 mol/L carbonate buffer (pH 9.6) and incubated at 37°C for 3 h or overnight at 4°C. The strips were blocked for 2 h at 37°C with 200μl of 15.0% skimmed milk prepared in PBS-Tween 20. Next, 100 μl of a 20-fold dilution of the plasma sample was added to the appropriate wells. The strips were subsequently placed in a 37°C incubator for 45 min. After washing four times with wash buffer (0.25% Tris base and 0.05% Tween 20), 100 μl of a 2,000-fold dilution of HRP-LD5 (1 mg/ml) was added to the strips and incubated for 45 min at 37°C. The strips were developed using 3, 3’, 5, 5’-tetramethylbenzidine (TMB) and a hydrogen peroxide mixture. The reaction was stopped by the addition of 2 M sulfuric acid, and the absorbance at 450 nm was read using an ELISA Reader (Thermo Scientific Multiskan Fc, Vantaa, Finland).

### Competitive inhibition ELISA

To estimate the contributions of full-length VP1 and the various truncated VP1 proteins to the anti-CA16 VP1 reaction, a competitive inhibition ELISA was performed as previously described [35, 40, 41]. Briefly, 96-well microtiter plates were coated with 1.0 μg of CA16 VP1 protein in 50 mM carbonate buffer (pH 9.6) overnight at 4°C and then blocked for 2 h at 37°C with 200 μl of 15.0% skimmed milk prepared in PBS-Tween 20. A 100 μl aliquot of a 20-fold dilution of each plasma sample with a high anti-VP1 antibody response from the two groups was pre-incubated with 2.0 μg of the inhibitor proteins (full-length VP1, VP1_41-297_, VP1_61-297_, VP1_1-60_, VP1_45-58_ and pET32a) for 1 h at 37°C; then, the samples incubated in the presence (test serum) or absence (serum control) of the inhibitor proteins were added to VP1-coated strips and incubated for 45 min at 37°C. After incubation, the plates were washed four times with wash buffer, followed by incubation with 100 μl of a 2,000-fold dilution of HRP-LD5 (1 mg/ml). The plates were incubated for 45 min at 37°C and then washed. The plates were developed using TMB and a hydrogen peroxide mixture. The reaction was stopped after suitable colour development by the addition of 2 M sulfuric acid, and the absorbance at 450 nm was read using an ELISA reader. Three parallel wells were measured for each test, and the mean of the absorbance from the three wells was used to calculate the percentage of inhibition (PI) as follows: PI = [100—(absorbance value of test serum—absorbance value of background) / (absorbance value of serum control—absorbance value of background) × 100)]. The background absorbance was obtained in the absence of sample or HRP-LD5.

### Neutralizing assay

Neutralization tests were performed in RD cells using a TCID50-reduction assay [19, 42]. Serum samples were incubated at 56°C for 30 min. Duplicate serum samples were diluted in Eagle’s MEM (Gibco) in a series ranging from 1:8 to 1:1024 dilutions. A total of 50 μl of 100 tissue culture infective doses (TCID50) of CA16 virus was mixed with 50 μl of the appropriate serum dilution and incubated at 37°C for two hours in the presence of CO_2_, followed by the addition of 100 μl of an RD cell suspension (1 x 10^5^ cells per 0.1 ml). Infected cells and controls were incubated at 37°C in 5.0% CO_2_ and observed with an inverted microscope daily for seven days. The highest dilution that prevented the development of cytopathic effects in 50.0% of the wells was considered the antibody titer of the sample. A virus back-titration was also performed to determine the virus titer and this serve as a no-serum control.

### Statistical analysis

Statistical analyses were performed using SPSS 17.0 and SAS 9.3 software. All experiments were performed in triplicate, and the values obtained from three replicate samples were averaged for each experiment. The statistical significance was tested using the Nemenyi non-parametric test, the Wilcoxon rank sum test or the independent samples t test. Differences between measurements were considered significant at p-values less than 0.05.

## Results

### Expression and purification of various CA16 VP1 proteins

**Fig 1:**
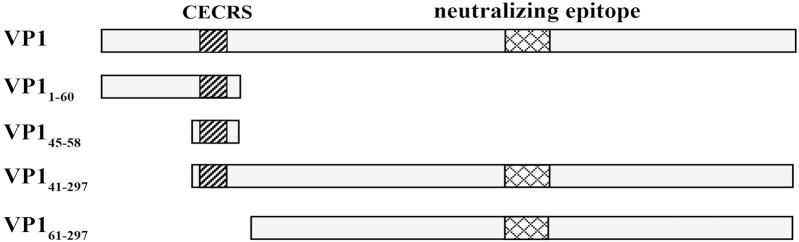
The design of the four truncated CA16 VP1 proteins. In the present study, four CECRS-based antigens VP1_1-297_ (full antigen), VP1_1-60_ (N antigen), VP1_45-58_ (core antigen), VP1_41-297_ (C antigen), and a control antigen (VP1_61-297_) without the CECRS were designed and constructed.

Our previous study demonstrated that the host antibody response against EV71 VP1 mainly targets epitopes based on the common enterovirus cross-reactive sequence (CECRS), and three types of antigens, including core antigen (VP1_45-58_), N antigen (VP1_1-60_) and C antigen (VP1_41-297_), were proposed [35]. In the present study, four CECRS-based antigens, CA16 VP1_1-297_ (full antigen), VP1_41-297_ (C antigen), VP1_1-60_ (N antigen), and VP1_45-58_ (core antigen), as well as a control antigen (VP1_61-297_) without the CECRS, were designed and constructed (Fig 1). The coding DNA for each of these antigens was separately amplified by PCR and inserted into a prokaryotic expression vector (pET32a or pET21b). Proteins were expressed in *E*. *coli* BL21 (DE3) cells and verified by SDS-PAGE. The size of each recombinant protein was in agreement with its expected molecular weight. pET21b-VP1_1-297_, pET32a-VP1_41-297_ and pET32a-VP1_61-297_ were located in inclusion bodies and could be easily solubilized in 8 M urea and conveniently purified under denaturing conditions. pET32a-VP1_1-60_ and pET32a-VP1_45-58_ were found in the soluble fractions of bacterial lysates. These soluble proteins were purified under native conditions.

### Characterization of antibody reactions to various CA16 VP1 antigens in Shanghai blood donors and Shanxi individuals

**Fig 2:**
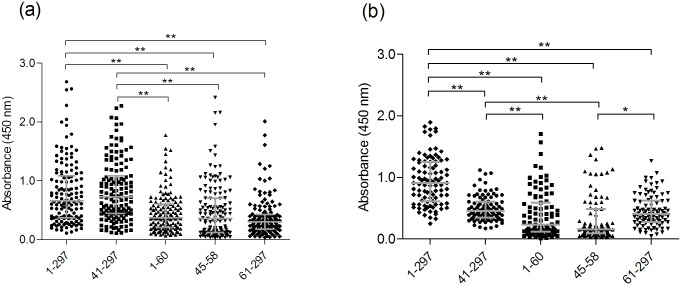
Comparison of the antibody reactivity against full-length and truncated VP1 proteins (CA16 VP1_41-297_, VP1_1-60_, VP1_45-58_, and VP1_61-297_) in 142 and 95 strongly VP1 reactive samples from the Shanghai blood donors (a) and Shanxi individuals (b), respectively. Each symbol represents an individual sample, and the lines indicate Q1, Q2 and Q3 value of the group from the bottom to the top, respectively. Statistical significance was tested using the Nemenyi non-parametric test. * indicates p<0.05, ** indicates p<0.001.

In total, 142 serum samples from Shanghai blood donors and 95 serum samples from Shanxi individuals were submitted to NEIBM-ELISA to determine the antibody reactivity of each against five CA16 VP1 antigens, namely, VP1_1-297_, VP1_41-297_, VP1_1-60_, VP1_45-58_ and VP1_61-297_. As shown in Fig 2a, the sample reactivity against the five antigens in the Shanghai group demonstrated two reactivity levels: high reactivity against VP1_1-297_ (full antigen) and VP1_41-297_ (C antigen) and low reactivity against VP1_1-60_ (N antigen), VP1_45-58_ (core antigen) and VP1_61-297_ (control antigen). In the Shanxi group, three reactivity levels were detected: high reactivity against VP1_1-297_, intermediate reactivity against VP1_41-297_ and VP1_61-297_, and low reactivity against VP1_1-60_ and VP1_45-58_ (Fig 2b). These results indicate obvious differences in the antibody responses between the two groups. Moreover, the reactivity level against VP1_41-297_ was significantly higher than that against VP1_61-297_ in the Shanghai group, indicating the importance of the CECRS in the antigenicity of VP1 antigens.

To further compare the antibody responses against CA16 VP1 between the two groups, correlation analyses among the antibody reactions against the five CA16 VP1 antigens were performed. As shown in S1a Fig and Table 1, the antibody reactions of the samples from the Shanghai group against each CA16 VP1 antigen were significantly correlated in all ten cases, even in the reactions against VP1_1-60_ and VP1_61-297_, which share no overlapping amino acid sequences. These correlation coefficients were generally higher than their equivalents in the Shanxi group, with the exception of two cases, namely, the reactions against VP1_1-297_ and VP1_1-60_ and the reactions against VP1_1-60_ and VP1_45-58_. In the Shanxi group, antibody reactions to the various CA16 VP1 antigens were significantly correlated in eight of ten cases (No. 1–8 in Table 1), and the correlation coefficients were usually lower than their equivalents in the Shanghai group (S1b Fig, Table 1). The reactions against VP1_1-60_ and VP1_61-297_, which share no overlapping amino acid sequences, were not significantly correlated. Furthermore, to our surprise, the reactions against VP1_41-297_ and VP1_61-297_, which share 237 overlapping amino acids, were also not significantly correlated in Shanxi individuals.

**Table 1:** Analysis of the correlation between the reactivity and inhibition of anti-CA16 VP1 reaction between complete CA16 VP1 and the four truncated VP1 proteins in the Shanghai blood donors and Shanxi individuals.

No.		Reactivity		Inhibition	
		Shanghai blood donors	Shanxi individuals	Shanghai blood donors	Shanxi individuals
1.	VP1_1-297_ *VS* VP1_41-297_	0.907**	0.331**	0.974**	0.958**
2.	VP1_1-297_ *VS* VP1_1-60_	0.631**	0.696**	0.914**	-0.021
3.	VP1_41-297_ *VS* VP1_1-60_	0.596**	0.334**	0.930**	-0.094
4.	VP1_1-297_ *VS* VP1_45-58_	0.791**	0.567**	0.550**	0.021
5.	VP1_41-297_ *VS* VP1_45-58_	0.784**	0.462**	0.562**	0.010
6.	VP1_1-60_ *VS* VP1_45-58_	0.541**	0.738**	0.670**	0.706**
7.	VP1_1-297_ *VS* VP1_61-297_	0.541**	0.316**	0.173	0.205
8.	VP1_45-58_ *VS* VP1_61-297_	0.547**	0.268**	0.291**	0.012
9.	VP1_41-297_*VS* VP1_61-297_	0.561**	0.096	0.234*	0.148
10.	VP1_1-60_ *VS* VP1_61-297_	0.235**	0.114	0.277*	0.086

**p<0.01,

*p<0.05

These results further demonstrated that the Shanghai group and the Shanxi group had different antibody reactions, with the individuals from Shanghai showing more correlated antibody reactions against the various CA16 VP1 antigens compared to the individuals from Shanxi.

### Characterization of the inhibitory effects of various antigens against anti-CA16 VP1 antibody responses in the two study populations

**Fig 3:**
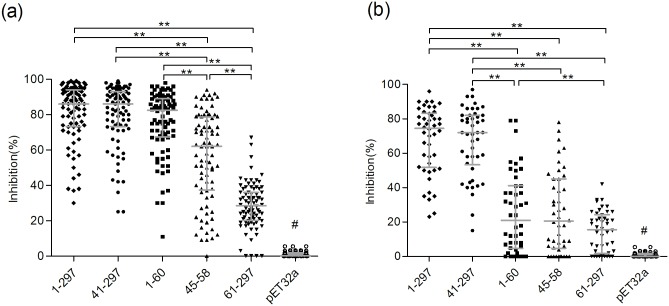
Comparison of the inhibition activities of five proteins in the Shanghai blood donors (a) and Shanxi individuals (b). The percentage of inhibition based on the competitive ELISA was plotted on the y-axis and six inhibitor proteins were plotted on the x-axis (CA16 VP1, VP1_41-297_, VP1_1-60_, VP1_45-58_, VP1_61-297_ and pET32a). Each symbol represents an individual sample, and the lines indicate Q1, Q2 and Q3 value of the group from the bottom to the top, respectively. Statistical significance was tested using the Nemenyi non-parametric test. * indicates p<0.05, ** indicates p<0.001. # represents p<0.05 comparing all other groups.

To further characterize the differences in antibody response against CA16 VP1 between the Shanghai blood donors and Shanxi individuals, 84 and 46 strongly reactive samples (serum samples were sorted based on the OD value of anti-CA16 VP1 reactivity, and the samples with the highest reactivity were chosen) from 142 Shanghai blood donors and 95 Shanxi individuals respectively, were submitted to competitive inhibition ELISA to determine the inhibition potencies of VP1_1-297_, VP1_41-297_, VP1_1-60_, VP1_45-58_ and VP1_61-297_. As shown in Fig 3a, in the Shanghai group, three significantly different levels of inhibition were produced by the various antigens: strong inhibition was produced by VP1_1-297_, VP1_41-297_ and VP1_1-60_ with more than 80.0% of the reaction inhibited; intermediate inhibition was produced by VP1_45-58_ with more than 60.0% of the reaction inhibited; and weak inhibition was produced by VP1_61-297_, with less than 30.0% of the reaction inhibited. In contrast, in the Shanxi group, only two significantly different levels of inhibition were identified: strong inhibition was produced by VP1_1-297_ and VP1_41-297_, with more than 70.0% of the reaction inhibited, and weak inhibition was produced by VP1_1-60_, VP1_45-58_ and VP1_61-297_, with less than 30.0% of the reaction inhibited (Fig 3b). Moreover, VP1_1-60_ inhibited more than 80% of the anti-VP1 reactions in the Shanghai group, whereas it inhibited less than 25% of the anti-VP1 reactions in the Shanxi group (Fig 3a and 3b). These results clearly demonstrate that the two study groups had different inhibition patterns for anti-CA16 VP1 and different antibody responses against CA16 VP1. In addition, all four CECRS-based antigens (VP1_1-297_, VP1_41-297_, VP1_1-60_ and VP1_45-58_) exhibited stronger inhibition potency than VP1_61-297_ in the Shanghai group (Fig 3a), and VP1_1-297_ and VP1_41-297_ exhibited stronger inhibition potency than VP1_61-297_ in the Shanxi group (Fig 3b), suggesting that anti-CA16 VP1 was mainly produced in response to CECRS-based epitopes in both groups (and especially the group from Shanghai), although there were different degrees of antibody production.

The correlation analysis of the CA16 VP1 inhibition produced by various VP1 antigens further demonstrated that the Shanghai and Shanxi groups showed different inhibition patterns. As shown in S2a Fig and Table 1, the inhibition of anti-CA16 VP1 produced by each pair of the five antigens was significantly correlated in all ten cases, with the exception of one case (inhibition by the pair VP1_1-297_ and VP1_61-297_), in the Shanghai blood donors. Moreover, the inhibition levels produced by each pair of the four CECRS-based antigens (Nos.1-6 in Table 1) in 6 of the cases showed higher correlations than the other four cases (inhibitions by the pair of VP1_61-297_ and each of four CECRS-based antigens). Specifically, the inhibition levels produced by the pairs of VP1_1-297_, VP1_41-297_ and VP1_1-60_ in the Shanghai group were very highly correlated, with correlation coefficients ranging from 0.914 to 0.974 (Nos.1-3 in Table 1). In contrast, the level of inhibition of the anti-CA16 VP1 reaction produced by each pair of the five antigens in the Shanxi group were significantly correlated in only two cases (by the pair VP1_1-297_ and VP1_41-297_ and the pair of VP1_1-60_ and VP1_45-58_). These results consistently demonstrated that different antibody responses occurred between the two study populations, and that the antibody responses against CA16 VP1 had higher correlations in the Shanghai group than the Shanxi group.

### The Shanghai and Shanxi groups showed different inhibition profiles against the anti-CA16 VP1 reaction

**Fig 4:**
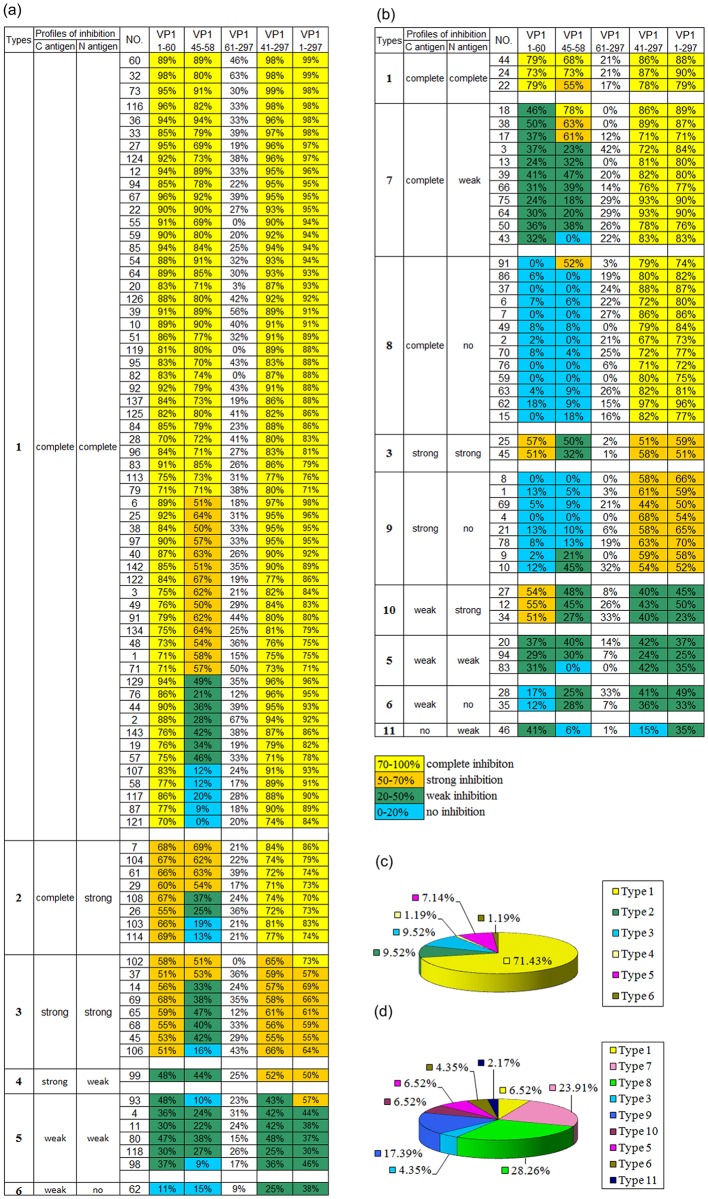
Characterization of different types of inhibition profiles against anti-CA16 antibody response by five antigens between the Shanghai blood donors (a) and Shanxi individuals (b). The inhibition profiles against anti-CA16 reaction were sorted based on the combination of inhibition degrees by VP1_1-60_ and VP1_41-297_. Eleven types of inhibition profiles were defined in both groups. Individual values are shown in colour as indicated beneath Fig 4b, with yellow representing the strongest inhibition degree. The constituent ratios of the types of inhibition profiles in the Shanghai blood donors (c) and Shanxi individuals (d) are shown.

As described above (Fig 3a), VP1_1-60_, the N antigen of VP1, exhibited the same strongest inhibition potency compared to VP1_41-297_ (the C antigen) and VP1_1-297_ (the full antigen) in Shanghai blood donors, whereas, in Shanxi individuals the inhibition potency of VP1_1-60_ was significantly lower than that of VP1_41-297_ or VP1_1-297_ (Fig 3b). Moreover, the levels of inhibition of the anti-CA16 VP1 reaction produced by VP1_1-60_ and VP1_41-297_ were strongly correlated in the Shanghai group, with a correlation coefficient greater than 0.9 (S2a Fig, Table 1). In contrast, these inhibitory potencies were not correlated (correlation coefficient of -0.094) in the Shanxi group (S2b Fig, Table 1). Considering that only 20 amino acids containing the CECRS overlapped between VP1_1-60_ and VP1_41-297_, the antigenicity between VP1_1-60_ and VP1_41-297_ should be the most distant among the four CECRS-based antigens. Based on these findings, we characterized the different inhibition profiles against anti-CA16 according to the degrees of inhibition produced by the N antigen and the C antigen. Eleven different inhibition profiles were defined among the Shanghai and Shanxi groups (Fig 4a and 4b). In the 84 strongly reactive samples from the Shanghai group, six different types of inhibition were found, including the following: 1) complete inhibition by the C antigen and the N antigen against full-length VP1; 2) complete inhibition by the C antigen and strong inhibition by the N antigen; 3) strong inhibition by the C antigen and the N antigen; 4) strong inhibition by the C antigen and weak inhibition by the N antigen; 5) weak inhibition by the C antigen and the N antigen; and 6) weak inhibition by the C antigen and no inhibition by the N antigen (Fig 4a). The inhibition type 1 accounted for 71.4% of the samples and was therefore the predominant inhibition type (Fig 4c).

In contrast to the above, in the 46 strongly reactive samples from the Shanxi group, nine different types of inhibition were identified, including types 1, 3, 5, and 6 described above in addition to the 5 following types (types 7–11): 7) complete inhibition by the C antigen and weak inhibition by the N antigen; 8) complete inhibition by the C antigen and no inhibition by the N antigen; 9) strong inhibition by the C antigen and no inhibition by the N antigen; 10) weak inhibition by the C antigen and strong inhibition by the N antigen; and 11) no inhibition by the C antigen and weak inhibition by the N antigen (Fig 4b). The main inhibition type in the Shanxi group was type 8, which accounted for 28.3% of the samples (Fig 4d), while inhibition type 1 accounted for only 6.5% of the samples in the Shanxi group vs. 71.4% in the Shanghai group. These results further demonstrated that the two study populations showed different antibody responses against CA16 VP1. It is very interesting, that inhibition type 1 was the predominant type of inhibition in the Shanghai group, and accounted for 71.4% of the strongly reactive samples from this group. Based on these results, we proposed that inhibition type 1 might represent specific antibody response.

### Characterization of neutralizing antibody responses in the two study groups

**Fig 5:**
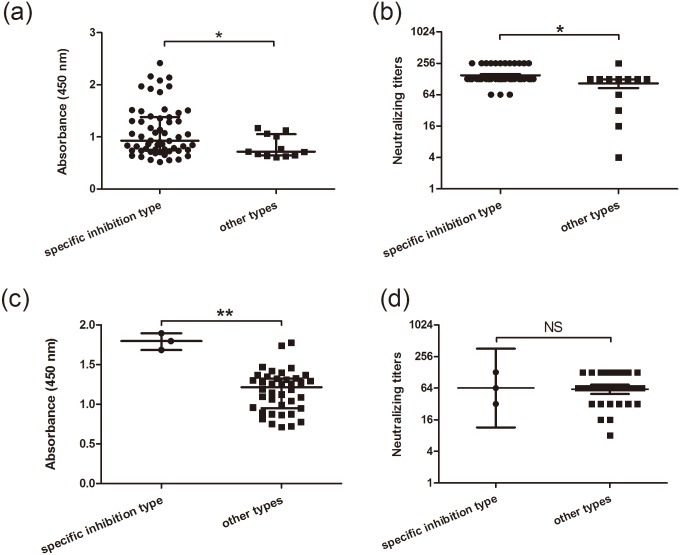
Characterization of antibody reactivity against CA16 VP1 and the anti-CA16 neutralizing titres of 70 and 43 serum samples strongly reactive against VP1 from the Shanghai blood donors (a and b) and Shanxi individuals (c and d). The samples strongly reactive against VP1 were divided into two groups: the specific inhibition type (the anti-CA16 VP1 reaction were completely inhibited by VP1_1-60_ and VP1_41-297_) and the other types. The specific inhibition type and the other types include 48 and 12 serum samples in the Shanghai blood donors and 3 and 40 serum samples in the Shanxi individuals, respectively. Statistical significance was tested using the independent samples t test and Wilcoxon rank sum test. * indicates p<0.05, ** indicates p<0.001, “NS” represents no significant difference (p>0.05).

To determine whether the inhibition type 1 might represent specific antibody response, the strongly reactive serum samples from both groups were submitted to the CA16 neutralizing antibody (NtAb) titer assays. Due to the limited volumes of the serum samples, only 70 (58 samples from inhibition type 1 and 12 samples from inhibition types 2–6) and 43 (3 samples from inhibition type 1 and 40 samples from inhibition types 3, 5, 6 and 7–11) serum samples that were strongly reactive against VP1 from the Shanghai and Shanxi groups, respectively, were utilized to detect anti-CA16 NtAb titers. For both groups, the samples with specific inhibition type showed significantly stronger reactivity compared to the samples with the other inhibition types (Fig 5a and 5c). The NtAb titers of the samples with the specific inhibition type were consistently significantly higher than those of the samples with the other inhibition types (Fig 5b) in the Shanghai group. In contrast, in the Shanxi group the NtAb titers of the samples with the specific inhibition type were not significantly higher than those of the samples with the other inhibition types (Fig 5d). This discrepancy might be because the sample number was small or due to the relatively low NtAb titers in the Shanxi group. These results consistently support that inhibition type 1 which was predominant in the Shanghai group could represent a specific antibody response against CA16.

## Discussion

In the current study, obvious differences were found not only in reaction and inhibition patterns against various CA16 VP1 antigens but also in correlations in reaction and inhibition patterns between the Shanghai and Shanxi groups (Figs 2 and 3, Table 1), suggesting that the two groups had different antibody responses against CA16 VP1. In theory, there should be two types of antibody response against the various CA16 VP1 antigens: responses elicited by CA16 infection (i.e., specific antibody responses) and responses elicited by infections of the many CA16-related enteroviruses (i.e., non-specific antibody responses). In the Shanghai group, only two levels of sample reactivity were identified (Fig 2a), and the antibody reactions against the various CA16 VP1 antigens were significantly correlated in all ten cases (S1a Fig). Additionally, the levels of inhibition of the anti-CA16 VP1 reaction produced by the five antigens were significantly correlated in nine out of ten cases, with comparably high correlation coefficients compared to their equivalents in the Shanxi group in most cases (S2 Fig, Table 1), suggesting that the antibody responses against CA16 VP1 in this group were simple and correlated. Conversely, in the Shanxi group, three levels of sample reactivity were identified (Fig 2b), and the antibody reactions of the samples against the various CA16 VP1 antigens were only significantly correlated in eight out of ten cases (S1b Fig). Furthermore, the levels of inhibition of the anti-CA16 VP1 reaction produced by the various antigens were only significantly correlated in two out of ten cases (S2b Fig, Table 1), suggesting that the antibody responses in this group were complex and less correlated. One explanation for higher correlation in antibody reaction and inhibition of anti-CA16 VP1 in the Shanghai group is that the antibodies in this group were mainly elicited by CA16 infection and therefore consistently reacted with various CA16 VP1 antigens, regardless of the presence of the CECRS, to generate correlated antibody reactions and inhibition of anti-CA16 VP1. Indeed, the reactions against VP1_1-60_ and VP1_61-297_ and the levels of inhibition of anti-CA16 VP1 produced by VP1_1-60_ and VP1_61-297_ were also significantly correlated in the Shanghai group, despite that these two antigens share no overlapping amino acid sequences (Table 1). In contrast, the antibody reaction in the Shanxi group were mainly elicited by infections with one or more CA16-related enteroviruses, and showed reactivity with each of the CA16 VP1 antigens in an inconsistent way, and hence generated the less correlated antibody reaction and inhibition of anti-CA16 VP1 (S1 and S2 Figs, Table 1).

In this study, the reactivity to VP1_1-60_ in the Shanghai group was significantly lower than the reactivity to VP1_41-297_ or VP1_1-297_ which produced similar levels of reactivity (Fig 2a). However, CA16 VP1_1-60_ produced similar and strong inhibition potency against anti-CA16 VP1 compared to VP1_1-297_ and VP1_41-297_ in the Shanghai group (Fig 3a). This result differs from a previous report on EV71 in which VP1_1-60_ and VP1_41-297_ showed significantly lower inhibition potency against anti-EV71 VP1 than VP1_1-297_ in severe HFMD cases [35]. Moreover, the inhibition potencies of VP1_1-60_, VP1_1-297_ and VP1_41-297_ were highly correlated, with correlation coefficients above 0.9 (S2a Fig, Table 1). This leads to the question of how VP1_1-60_ could inhibit anti-CA16 VP1 as effectively as VP1_1-297_ and VP1_41-297_, which contain the whole and most of the VP1 amino acid sequence, respectively. One explanation is that the CECRS plays a particularly important role in the antigenicity of CA16 VP1 antigens compared to EV71 VP1 antigens, and that large amounts of N antigen (VP1_1-60_) could effectively inhibit interactions between specific antibodies and CECRS-containing VP1 antigens including VP1_1-297_ and VP1_41-297_. This inhibition would in turn prevent the binding of specific antibodies to all CECRS-based epitopes in VP1. Consistently, the core antigen (VP1_45-58_) exhibited strong inhibition potency similar to that produced by VP1_1-60_ (Fig 3a).

We characterized the anti-CA16 VP1 reaction based on the degrees of inhibition produced by VP1_1-60_ and VP1_41-297_ (two CECRS-based antigens with substantial differences in amino acid sequence) to define 11 different inhibition profiles for this reaction (Fig 4). The Shanghai group showed 6 different inhibition types, including the a specific inhibition type (type 1), which was predominant in this group, and accounted for 71.4% of the samples. In contrast, the Shanxi group showed 9 inhibition profiles, and type 8 was predominant, accounting for 28.3% of the samples (Fig 4d), while type 1 accounted for only 6.5% of the samples. In the Shanghai group, the neutralizing antibody titers in the samples with inhibition type 1 were consistently significantly higher than those in the samples with the other inhibition types, indicating that CA16 infection was common in this group (Fig 5b). These findings support the conclusion that the anti-CA16 VP1 responses observed in most of (71.4%) the Shanghai group could be attributed to specific CA16 infection, whereas the antibody responses in most of (93.5%) the Shanxi group could be attributed to infections with other CA16-related enteroviruses. Whether the different types of inhibition identified here were elicited by infections with different types of enterovirus remains to be elucidated. Our previous results revealed that antibody responses against EV71 VP1 are predominantly targeted to epitopes containing the CECRS which is highly conserved among different enteroviruses [35]. A 9-amino acid sequence of the CECRS in CA16 VP1 is identical to sequences found in most enteroviruses in *EV A* species, except for that in EV71 VP1, in which the sequence contains one amino acid mutation, and is highly conserved among six other species of enteroviruses (enteroviruses B, C, D and rhinovirus A, B and C) that infect humans with two or three amino acid mutations. Hence, CA16 VP1 could cross-react with antibodies elicited by enteroviruses from seven species that infects humans at different levels of prevalence. Consistently, 11 different inhibition profiles were found in the two study groups, with the Shanghai and Shanxi groups showing 6 and 9 types, respectively. These different inhibition profiles were assumed to arise from infections with different enteroviruses, suggesting that broad cross reactivity against CA16 VP1 can be produced from many CA16-related enteroviruses.

Intriguingly, although VP1_41-297_ contained a 257 amino acid sequence that overlapped with most of VP1_1-297_, the correlations between the anti-VP1_41-297_ and anti-VP1_1-297_ reactions in the Shanghai and Shanxi groups varied greatly (Table 1). The anti-VP1_41-297_ and anti-VP1_1-297_ responses in the Shanghai group were highly correlated, with correlation coefficients above 0.9. In contrast, in the Shanxi group, these two reactions were correlated at a very low level, with a correlation coefficient of 0.331. One explanation for this difference is as follows. The antibody responses against the various CA16 VP1 antigens in the Shanghai group was assumed to arise from infection with CA16. In this case, antibodies produced against VP1_41-297_ and VP1_1-297_ should be homologous, resulting in strongly correlated reactions. In contrast, the antibody responses in the Shanxi group were assumed to be elicited by infections with other CA16-related enteroviruses. In this case, antibodies produced against VP1_41-297_ and VP1_1-297_ should be heterologous, leading to less correlated reactions. From this viewpoint, the correlation coefficients of reactions against VP1_41-297_ and VP1_1-297_ could be an effective index for evaluating the degree of consistency among anti-CA16 VP1 reactions, with high correlation coefficients indicating the degree of epidemic of a specific CA16 infection. Moreover, this finding suggests that it is feasible to use an ELISA approach to detect specific antibodies against CA16 VP1 by using CA16 VP1_41-297_ as a coating antigen and the VP1_41-297_ from one or more viruses closely related to CA16 as inhibitors to eliminate non-specific antibody responses. In addition, the obvious differences in the reaction correlations between the Shanghai and Shanxi groups were evident in multiple comparisons: VP1_41-297_ verses VP1_1-60_, VP1_1-297_ verses VP1_45-58_, VP1_41-297_ verses VP1_45-58_, VP1_1-297_ verses VP1_61-297_, VP1_45-58_ verses VP1_61-297_, VP1_41-297_ verses VP1_61-297_, and VP1_1-60_ verses VP1_61-297_ (Table 1). Moreover, differences between the Shanghai and Shanxi groups were also obvious in almost all of the inhibition correlation results, with the exception of two cases: VP1_1-297_ verses VP1_41-297_ and VP1_1-60_ verses VP1_45-58_ (Table 1). When considering the differences among these correlations, the three following cases were particularly striking: the correlation coefficient for the reaction between anti-VP1_41-297_ and anti-VP1_1-297_ was above 0.9 in the Shanghai group, compared to 0.331 in the Shanxi group. Furthermore, the correlation coefficients of inhibition between VP1_1-297_ verses VP1_1-60_ and VP1_41-297_ verses VP1_1-60_ were both above 0.9 in the Shanghai group compared to -0.021 and -0.094 in the Shanxi group (Table 1). How these differences might reflect the specific epidemicity of a specific CA16 infection remains unknown, however, they might provide a potential basis for identifying an effective approach for performing seroepidemiological surveillance of CA16 infections based on ELISA.

Our previous study revealed that human anti-EV71 antibody responses predominately target VP1, particularly the CECRS-based epitopes on VP1 [35]. In this study, reactivity to VP1_41-297_ was significantly higher than reactivity to VP1_61-297_ (Fig 2a), and all of the CECRS-based antigens (VP1_1-297_, VP1_41-297_, VP1_1-60_ and VP1_45-58_) showed significantly higher inhibition potency than VP1_61-297_ in the samples from the Shanghai group (Fig 3a). These results indicate that the antibody responses against CA16 VP1 are equivalent to response against EV71 VP1, which also predominantly occur in response to CECRS-based epitopes. In the Shanxi group, although the reactivity against VP1_41-297_ was not significantly different from that against VP1_61-297_ (Fig 2b), the inhibition of VP1_41-297_ was significantly higher than that of VP1_61-297_ (Fig 3b), suggesting that the CECRS played a main role in the antibody responses against CA16 VP1 that were measured in the Shanxi group.

Our original study aim was to characterize antibody responses against CA16 VP1 at different times in blood donors from Shanghai since May, 2012. This would be helpful to distinguish the specific CA16 infection from the other Enterovirus infections in blood donors at different times. However, no significant differences had been found. The obviously different antibody response against CA16 VP1 between the Shanxi and Shanghai groups was an accidental finding that arose when the Shanxi specimens were used as a control. The Shanghai group specimens with a mean age of 35.5 years old were collected from blood donors from 17 July to 31 July 2013 in a region of Shanghai situated in eastern China. In contrast, the Shanxi group specimens with mean age of 61.2 years old were collected from 29 May to 30 May, 2013 in Taiyuan, which is situated in central China. The obvious differences in non-neutralizing antibody responses against CA16 VP1 between the two groups could therefore be caused by differences in age, host genetic factors and past coxsackievirus viral infections. How these other factors affected the antibody responses in these cases remain to be investigated.

In summary, our results demonstrated that individuals from Shanghai and Shanxi show different antibody responses against CA16 VP1. A potentially consistent and specific antibody response was found in the Shanghai blood donors, whereas an inconsistent and non-specific response was observed in the Shanxi individuals. These findings may improve understanding of host humoral immunity against CA16 and help identify an effective approach that can be used for seroepidemiological surveillance and specific diagnosis of CA16 infection based on normal and competitive ELISA.

## Supporting Information

S1 Fig Correlation analysis of the antibody reaction against various antigens in the Shanghai blood donors (a) and Shanxi individuals (b). The correlation was assessed using Spearman’s correlation coefficient. Correlation coefficient values (r), p values and the sample sizes (n) are shown.
(TIF)

S2 Fig Correlation analysis of inhibition of CA16 VP1 by various antigens in the Shanghai blood donors (a) and Shanxi individuals (b). The correlation was assessed using Spearman’s correlation coefficient. Correlation coefficient values (r), p values and the sample sizes (n) are shown.
(TIF)

S1 Table Baseline characteristics of the study participants. (DOCX)

S2 Table Primers for amplifying full-length and truncated CA16 VP1. (DOCX)
