# Supplementary figures and images for: Different Antibody Response against the Coxsackievirus A16 VP1 Capsid Protein: Specific or Non-Specific
Source: PLoS One. 2016 Sep 13;11(9):e0162820. doi: 10.1371/journal.pone.0162820 (PMC5021329; doi:10.1371/journal.pone.0162820)

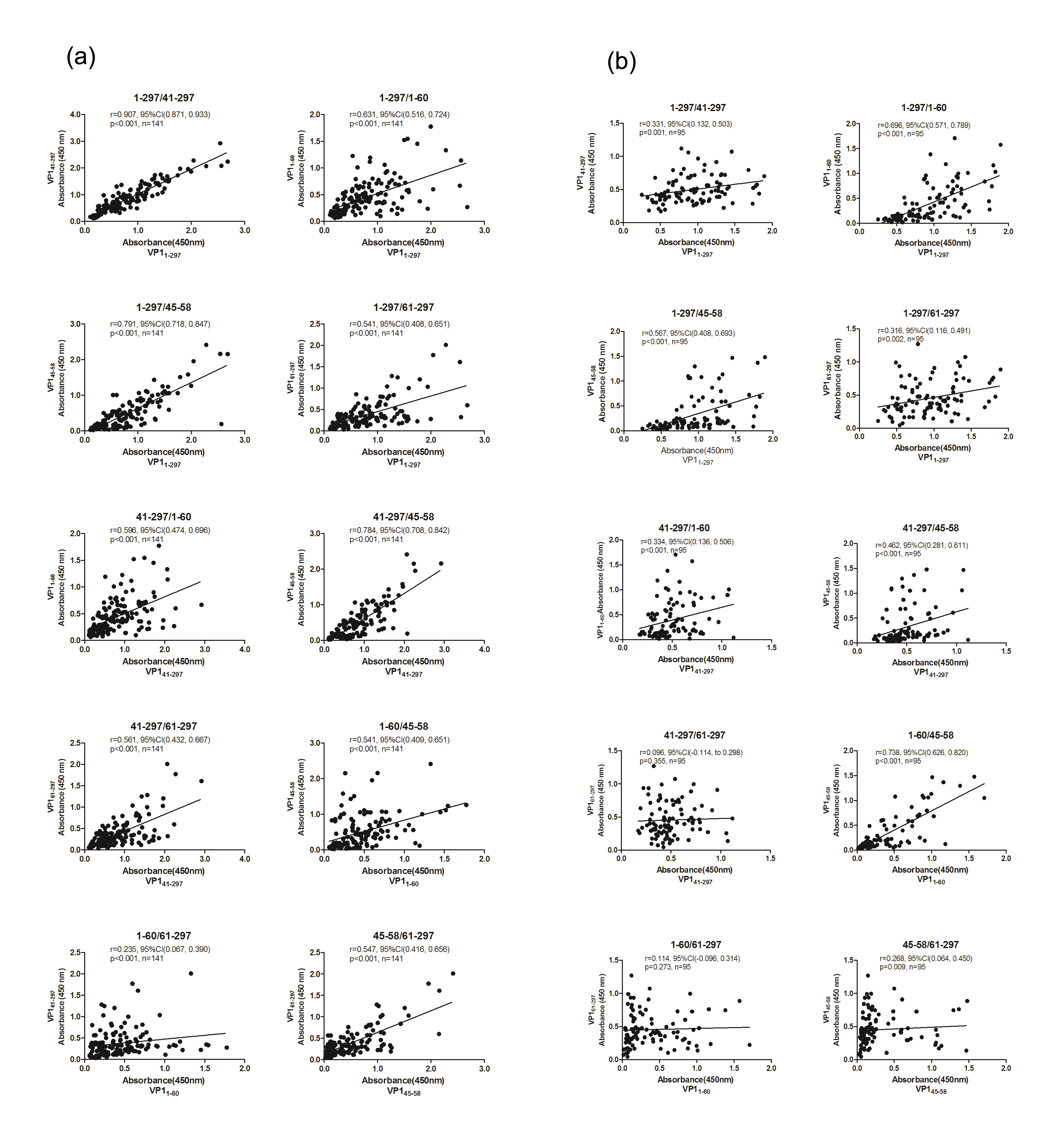

Supplement: S1 Fig — The correlation was assessed using Spearman’s correlation coefficient. Correlation coefficient values (r), p values and the sample sizes (n) are shown. (TIF) [file pone.0162820.s001.tif]

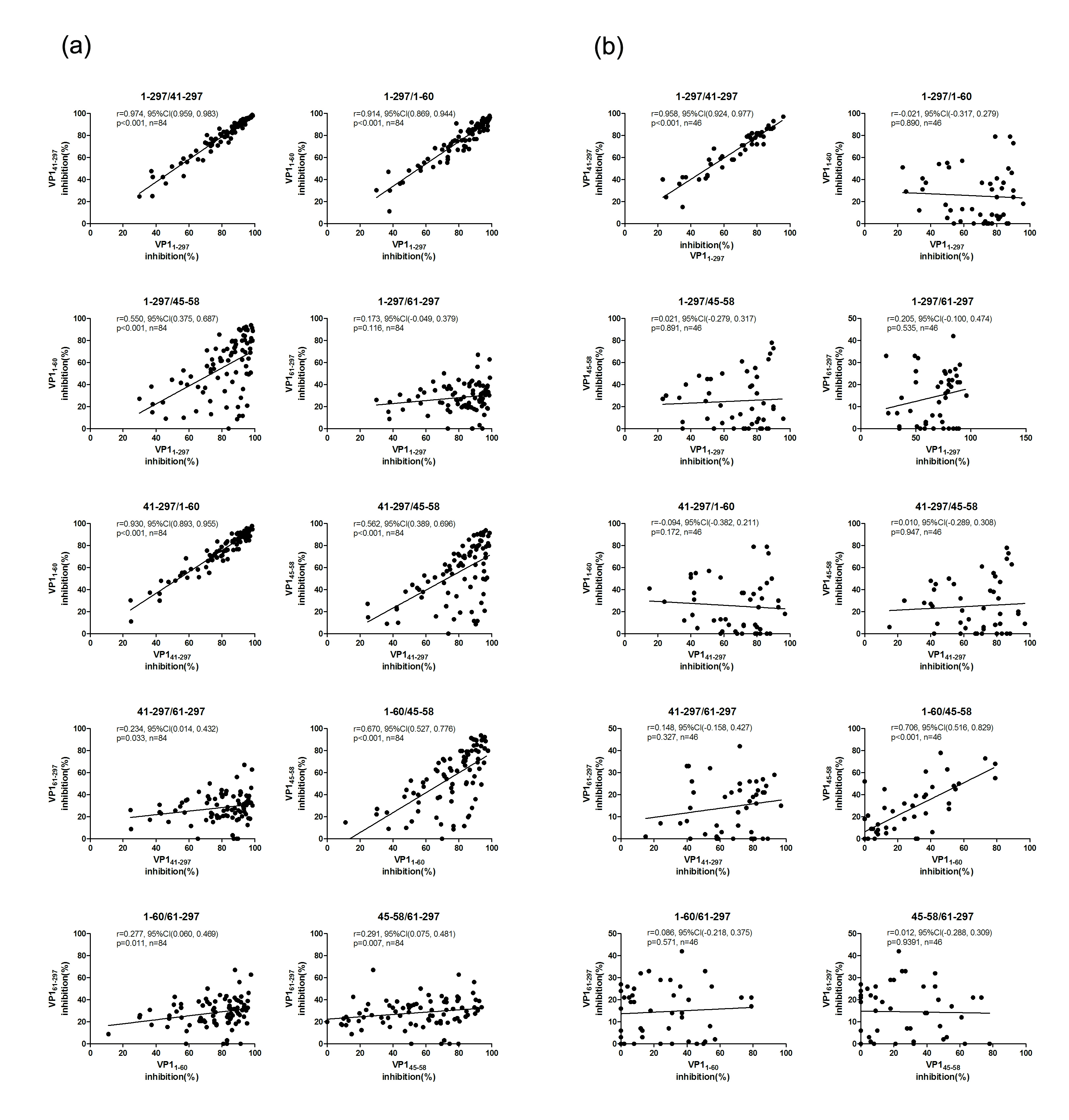

Supplement: S2 Fig — The correlation was assessed using Spearman’s correlation coefficient. Correlation coefficient values (r), p values and the sample sizes (n) are shown. (TIF) [file pone.0162820.s002.tif]
