# Supplementary material for: Different Antibody Response against the Coxsackievirus A16 VP1 Capsid Protein: Specific or Non-Specific
Source: PLoS One. 2016 Sep 13;11(9):e0162820. doi: 10.1371/journal.pone.0162820 (PMC5021329; doi:10.1371/journal.pone.0162820)
Supplement: S1 Table — (DOCX) [file pone.0162820.s003.docx]

|  | Shanxi Individuals | Shanghai Blood Donors |
| --- | --- | --- |
| **Area** | Taiyuan, Shanxi, China | Shanghai, China |
| **Number^a^** | 95 | 142 |
| **Time** | 2013.5.29-5.30 | 2013.7.17-7.31 |
| **Age (yr)** |  |  |
| Mean ± s.d.^b^ | 61.2 $\pm$ 14.0 | 35.5 $\pm$ 11.2 |
| Range | 19-87 | 17-56 |
| **Sex (%)** |  |  |
| Male | 50.5% | 53.5% |
| Female | 49.5% | 46.5% |

**S1 Table** Baseline characteristics of the study participants

^a^Number of evaluable individuals; ^b^Standard deviation;
