# Supplementary material for: Different Antibody Response against the Coxsackievirus A16 VP1 Capsid Protein: Specific or Non-Specific
Source: PLoS One. 2016 Sep 13;11(9):e0162820. doi: 10.1371/journal.pone.0162820 (PMC5021329; doi:10.1371/journal.pone.0162820)
Supplement: S2 Table — (DOCX) [file pone.0162820.s004.docx]

**S2 Table** Primers for amplifying full-length and truncated CA16 VP1

| Primers | Sequences (5’- 3’) | Description |
| --- | --- | --- |
| uVP1 | GCCGCGCATATG GGTGACCCGATCGCTGAC | The primer pairs uVP1/dVP1 were used to amplify VP1. uVP1 contains *Nde* I restriction sites (underlined), dVP1 contains *Xho* I restriction sites (underlined). |
| dVP1 | GCCGGCAAGCTT CAGAGTAGTGATTTTGTC |  |
| uVP1-1 | GCCGCGCCATGG CTACTGGTGTTGTTCCGGCT | The primer pairs uVP1-1/dVP1-1 and uVP1-2/dVP1-1 were used to amplify VP1_41-297_ and VP1_61-297_. uVP1-1 and uVP1-2 contain *Nco* I restriction sites (underlined), dVP1-1 contain *Sac* I restriction sites (underlined). |
| dVP1-1 | GCCGGCGAGCTCTTA CAGAGTAGTGATTTTGTC |  |
| uVP1-2 | GCCGCGCCATGG CTAAAAACCTGATCGAAACT |  |
| uVP1-3 | GCCGCGGGATCC GGTGACCCGATCGCTGACATG | The primer pairs uVP1-3/dVP1-3 and uVP1-4/dVP1-4 were used to amplify VP1_1-60_ and VP1_45-58_. uVP1-3 and uVP1-4 contain *Bam*H I restriction sites (underlined), dVP1-1 contain *Hin*d III restriction sites (underlined). |
| dVP1-3 | GCCGGCAAGCTTTTA GTCAGAAGCGTTAGAAGA |  |
| uVP1-4 | GCCGCGGGATCC CCGGCTCTGCAGGCTGCT |  |
| dVP1-4 | GCCGGCAAGCTTTTA AGCGTTAGAAGAAGCACC |  |
